# Supplementary material for: How Much Do Front-Of-Pack Labels Correlate with Food Environmental Impacts?
Source: Nutrients. 2023 Feb 26;15(5):1176. doi: 10.3390/nu15051176 (PMC10005439; doi:10.3390/nu15051176)
Supplement: Supplementary file 1 [file nutrients-15-01176-s001.zip › Table S1.pdf]

Table S1. List of selected food items divided by food category, their nutrient composition, and their source on the BDA-IEO Database.

Food Items	Energy (KJ)	Total fats (g)	SFAs (g)	Sugars (g)	Proteins (g)	Salt (g)	Fibers (g)	Sodium (mg)	Food Category	Source on BDA-IEO Database
BREAD MULTICEREAL	946	1.1	0.15	9.9	6.2	1.38	4.6	553	Bakery products	Pane di Grano e Segale
BREAD PLAIN	1258	0.4	0.1	1.9	8.2	1.60	3	640	Bakery products	Pane 00
BREAD WHOLE	1082	1.3	0.3	2.5	7.5	1.38	5.7	550	Bakery products	Pane Integrale
CAKES AND CROISSANT	1754	18.3	10.2	10.6	7.2	0.98	2.5	390	Bakery products	Brioche o Croissant
CHOCOLATE OR CREAM FILLED COOKIES	2220	27.6	5.55	43.4	5.7	0.40	2.9	160	Bakery products	Biscotti
COCOA CAKES AND CROISSANT	1348	17.5	6.97	27.6	4.9	0.26	3.6	103	Bakery products	Torta al cioccolato
FLAVORED CRACKERS	2126	25.5	9.45	0	9.8	2.49	2.8	995	Bakery products	Crackers al formaggio
SIMPLE COOKIES	1776	8.5	1.7	35.7	13.8	1.23	1.2	491	Bakery products	Biscotti al latte
WHOLEGRAIN CRACKERS	1800	17.2	3.08	0	8.8	1.65	10.5	659	Bakery products	Crackers integrali
EGGS	533	8.7	3.17	0	12.4	0.34	0	137	Eggs	Uova
ANCHOVY	406	2.6	1.3	1.5	16.8	0.26	0	104	Fish and Seafood	Acciughe
ANGLERFISH	264	1	0.25	0.6	12.8	0.05	0	18	Fish and Seafood	Rana Pescatrice
CARP	584	7.1	1.37	0	18.9	0.13	0	50	Fish and Seafood	Carpa
CATFISH	389	3.3	0.84	0.2	15.5	0.16	0	63	Fish and Seafood	Pesce gatto
COD	300	0.3	0.06	0	17	0.19	0	77	Fish and Seafood	Merluzzo
COD FISH STICK	801	10.1	1.85	0.6	11	1.20	0	480	Fish and Seafood	Bastoncini di merluzzo
CRUSTACEANS	303	0.8	0.15	1.7	14.4	0.55	0	220	Fish and Seafood	Crostacei NS
CUTTLEFISH	305	1.5	0.43	0.7	14	0.24	0	97	Fish and Seafood	Seppie
EEL	1032	21.65	5.82	0.4	13.2	0.19	0	76	Fish and Seafood	Average of Anguilla di mare e di fiume †
FISH MIXED	444	3.7	0.97	0.7	17.4	0.21	0	82	Fish and Seafood	Pesce NS, con lisca
FLATFISH	439	2.3	0.33	0	20.8	0.14	0	54	Fish and Seafood	Halibut
HERRING	898	16.7	3.33	0	16.5	0.38	0	150	Fish and Seafood	Aringa
LOBSTER	358	1.9	0.63	1	16	0.44	0	177	Fish and Seafood	Aragosta
MACKEREL	708	11.1	2.61	0.5	17	0.33	0	130	Fish and Seafood	Maccarello
MUSSELS	353	2.7	0.52	0.3	11.7	0.73	0	290	Fish and Seafood	Cozze, o Mitili
OCTOPUS	240	1	0.42	1.4	10.6	0.58	0	233	Fish and Seafood	Polpo
POMFRET	508	3.8	0.88	1	20.7	0.21	0	84	Fish and Seafood	Orata, selvaggia
PRAWNS/SHRIMPS	300	0.6	0.09	2.9	13.6	0.37	0	146	Fish and Seafood	Gambero
RHOMBUS	344	1.3	0.17	1.2	16.3	0.21	0	83	Fish and Seafood	Rombo
ROCK FISH	347	0.4	0.09	0.6	19	0.13	0	53	Fish and Seafood	Scorfano

SALMON	773	12	2.97	1	18.4	0.25	0	98	Fish and Seafood	Salmone
SARDINE	544	4.5	1.02	1.5	10.8	0.17	0	66	Fish and Seafood	Sarda
SEA BASS	346	1.5	0.35	0.6	16.5	0.17	0	69	Fish and Seafood	Spigola ICA
SHARK	337	1.2	0.25	1.3	16	0.30	0	120	Fish and Seafood	Palombo
SNAILS	544	0.4	0.03	0	23.8	0.52	0	206	Fish and Seafood	Lumache di mare
SOLE	363	1.7	0.22	0.8	16.9	0.30	0	120	Fish and Seafood	Sogliola
SQUID	287	1.7	0.85	0.6	12.6	0.46	0	185	Fish and Seafood	Calamaro
SWORDFISH	459	4.2	1.15	1	16.9	0.23	0	90	Fish and Seafood	Pesce spada
TROUT	361	3	0.7	0	14.7	0.10	0	40	Fish and Seafood	Trota
TUNA	667	8.1	3.35	0.1	21.5	0.11	0	43	Fish and Seafood	Tonno
APPLE	184	0	0	10	0.2	0.01	2.6	3	Fruits	Mele, con buccia
APRICOT	131	0.1	0.01	6.8	0.4	0.00	1.5	1	Fruits	Albicocche
AVOCADO	981	23	2.48	1.8	4.4	0.01	3.3	2	Fruits	Avaocado
BANANA	292	1.2	0.11	12.8	1.2	0.00	1.8	1	Fruits	Banana
BLACKBERRY	177	0	0	8.1	1.3	0.01	3.2	2	Fruits	More
BLUEBERRIES	129	0.2	0.02	5.1	0.9	0.01	3.1	2	Fruits	Mirtilli
CHERRY	172	0.1	0.02	9	0.8	0.01	1.3	3	Fruits	Ciliegie
CLEMENTINE	243	0.2	0.03	12.8	0.8	0.01	2.2	2	Fruits	Mandaranci e Clementine
CURRANTS	165.5	0	0	5.5	1	0.01	7.6	2.5	Fruits	Average of Ribes nero e rosso †
DATES	544	0.1	0.01	31.3	1.5	0.02	1.8	7	Fruits	Datteri
FIG	218	0.2	0.04	11.2	0.9	0.01	2	2	Fruits	Fichi
GRAPEFRUIT AND POMELO	122	0	0	6.2	0.6	0.00	1.6	1	Fruits	Pompelmo
GRAPES	274	0.1	0.03	15.6	0.5	0.00	1.5	1	Fruits	Uva
GUAVA	130	0.5	0.14	3.5	0.7	0.01	5.4	4	Fruits	Guava
KIWI	204	0.6	0.03	9	1.2	0.01	2.2	5	Fruits	Kiwi
LEMON	62	0	0	2.3	0.6	0.01	1.9	2	Fruits	Limone
LIME	190	0.3	0.03	8.8	0.7	0.01	3.3	2	Fruits	Limette
MANDARIN	322	0.3	0.04	17.6	0.9	0.00	1.7	1	Fruits	Mandarini
MANGO	239	0.2	0.05	12.6	1	0.00	1.6	1	Fruits	Mango
MELON	100	0.2	0.05	4.9	0.5	0.08	0.7	32	Fruits	Melone invernale
ORANGE	157	0.2	0.03	7.8	0.7	0.01	1.6	3	Fruits	Arance
PEACH	128	0.1	0.01	6.1	0.8	0.01	1.6	3	Fruits	Pesche
PEAR	180	0.1	0.02	8.8	0.3	0.01	3.8	2	Fruits	Pere
PINEAPPLE	176	0	0	10	0.5	0.01	1	2	Fruits	Ananas
PLUM	192	0.1	0.01	10.5	0.5	0.01	1.5	2	Fruits	Prugne

POMEGRANATE	288	0.2	0.02	15.9	0.5	0.02	2.2	7	Fruits	Melagrane
QUINCES	157	0.1	0.01	6.3	0.3	0.01	5.9	3	Fruits	Mele cotogne
RAISIN	1248	0.6	0.08	72	1.9	0.13	5.2	52	Fruits	Uva Sultanina, Uvetta, Uva secca
RASPBERRIES	202	0.6	0.02	6.5	1	0.01	7.4	3	Fruits	Lamponi
ROCKMELON	145	0.2	0.05	7.4	0.8	0.02	0.7	8	Fruits	Melone Estivo
STRAWBERRIES	128	0.4	0.02	5.3	0.9	0.01	1.6	2	Fruits	Fragole
TANGERIN	243	0.2	0.03	12.8	0.8	0.01	2.2	2	Fruits	Mandaranci e Clementine
WATERMELON	68	0	0	3.7	0.4	0.01	0.2	3	Fruits	Cocomero
BARLEY	1429	1.4	0.29	0	10.4	0.01	9.1	3	Grains	Orzo
CORNFLAKES	1582	0.8	0.13	10.4	6.6	0.03	3.8	11	Grains	Fiocchi di mais
EGG PASTA	1339	2.8	0.79	1.7	11.8	0.07	2.9	27	Grains	Pasta all'uovo, fresca
MAIZE	1533	3.8	0.48	2.5	9.2	0.09	2.9	35	Grains	Mais
OAT	1519	6.9	1.22	2.8	16.9	0.01	10.6	2	Grains	Avena
OAT MEAL	1656	7.5	1.32	0	8	0.08	8.3	33	Grains	Fiocchi d'avena
PASTA	1540	0.3	0.07	2.7	10.8	0.01	2.6	5	Grains	Pasta di Semola
PLAIN CRACKERS	1834	10	1.92	2.6	9.4	2.20	2.8	879	Grains	Crackers, salati
RICE	1554	0.6	0.14	0.2	7	0.02	1.4	6	Grains	Riso, brillato
RYE	1521	2	0.23	0	8.2	0.00	11.7	1	Grains	Farina di Segale, integrale
BEEF BONE FREE MEAT	524.6	4.64	1.658	0	20.82	0.16	0	62.2	Meats	Average of Vitello and Vitellone values (Grassa, Semigrassa, Magra) †
CHICKEN BONE FREE MEAT	463	3.6	1.23	0	19.4	0.18	0	70	Meats	Pollo intero, senza pelle
DUCK MEAT BONE FREE	667	8.2	2.54	0	21.4	0.28	0	110	Meats	Anatra
LAMB BONE FREE MEAT	848.3	14.2	6.91	0	19	0.25	0	100	Meats	Average of Agnello (Grassa, Semigrassa, Magra) †
PORK BONE FREE MEAT	850	14.45	4.99	0	18.55	0.17	0	67.5	Meats	Average of Suino (Grassa e Semigrassa) †
POULTRY MEAT	530.3	4.73	1.56	0	20.9	0.21	0	85	Meats	Average of Chicken, Turkey and Duck §
RABBIT MEAT WITH BONE	497	4.3	1.7	0	19.9	0.17	0	67	Meats	Coniglio,intero
TURKEY MEAT BONE FREE	461	2.4	0.9	0	21.9	0.19	0	75	Meats	Tacchino intero, senza pelle
ALMOND/COCONUT MILK	614.5	13.55	10.71	4.35	1.8	0.02	1.5	8	Milk Alternatives	Average of Almond and Coconut Milk §
ALMOND MILK	239	3.3	0.28	5.4	1.3	0.00	0.8	1	Milk Alternatives	Latte di Mandorla
COCONUT MILK	990	23.8	21.14	3.3	2.3	0.04	2.2	15	Milk Alternatives	Latte di cocco
SOYMILK	132	1.9	0.21	0.8	2.9	0.08	0	32	Milk Alternatives	Latte di soia
ASIAGO	1494	25.6	15	0.8	31.4	1.90	0	760	Milk and Dairys	Asiago
BUFFALO MILK	426	7.3	4.87	5.1	4.4	0.13	0	52	Milk and Dairys	Latte di Bufala
CAMEMBERT	1232	23.7	14.91	0	20.9	1.63	0	650	Milk and Dairys	Camembert
CEDDAR	1580	31	18.52	0.5	25	1.53	0	610	Milk and Dairys	Cheddar

CHEESE	1382.5	26.5	15.8	1.2	22.5	1.57	0.0	627.1	Milk and Dairys	Average of all listed cheeses §
CHEESE SEMI-HARD	1553	28.2	16.69	2	28.1	2.15	0	860	Milk and Dairys	Provolone
CREAM	1205	30	17.46	3.2	2.6	0.09	0	34	Milk and Dairys	Panna 30% di lipidi
EMMENTAL	1674	30.6	17.83	3.6	28.5	1.13	0	450	Milk and Dairys	Emmenthal
GOAT CHEESE	739	14.3	9.1	0.5	11.9	2.47	0	988	Milk and Dairys	Cacioricotta di capra
GOAT MILK	319	4.8	3.32	4.7	3.9	0.10	0	40	Milk and Dairys	Latte di capra
GRANA PADANO	1690	28.5	17.53	3.7	33.9	1.75	0	700	Milk and Dairys	Grana
MASCARPONE	1873	47	27.55	0.3	7.6	0.22	0	86	Milk and Dairys	Mascarpone
MILK	264	3.6	2.11	4.7	3.3	0.12	0	47	Milk and Dairys	Latte intero, UHT
MOZZARELLA	1051	19.5	11.43	0.7	18.7	0.50	0	200	Milk and Dairys	Mozzarella
PARMIGIANO REGGIANO	1609	28.1	18.54	0	33.5	1.39	0	556	Milk and Dairys	Parmigiano
PECORINO	1626	32	17.34	0.2	25.8	4.50	0	1800	Milk and Dairys	Pecorino
RICOTTA	609	10.9	6.82	3.5	8.8	0.20	0	78	Milk and Dairys	Ricotta
STRACCHINO	1243	25.1	14.71	0	18.5	1.04	0	414	Milk and Dairys	Stracchino
YOGHURT	278	3.9	2.07	4.3	3.8	0.12	0	48	Milk and Dairys	Yogurt di latte intero
YOGURT FLAVOURED	373	3.3	0.71	12.6	2.8	0.12	0.2	48	Milk and Dairys	Yogurt di latte intero alla frutta zuccherato
ALMOND	2595	55.3	4.59	3.7	22	0.04	12.7	14	Nuts and seeds	Mandorle secche
ALMOND PASTE	2017	25.8	2.14	54.3	8	0.01	7.2	4	Nuts and seeds	Pasta di Mandorle
CARROB	1065	0.7	0.1	48.9	3.3	0.09	23.1	35	Nuts and seeds	Carrube
CASHEW NUT	2282	46	8.17	5.6	15	0.04	3	16	Nuts and seeds	Anacardi
CHESTNUTS	737	1.7	0.3	8.9	2.9	0.02	4.7	9	Nuts and seeds	Castagne
HAZELNUT	2769	64.1	4.16	4.1	13.8	0.03	8.1	11	Nuts and seeds	Nocciole
NUTS	2617.3	56.6	5.9	4.0	18.7	0.02	8.6	8.0	Nuts and seeds	Average of Almond, Hazelnut, Walnut, Pistachio, cashew, Peanut §
PEANUT	2566	50	7.13	3.1	29	0.02	10.9	6	Nuts and seeds	Arachidi
PISTACHIO	2598	56.1	5.61	4.5	18.1	0.00	10.6	1	Nuts and seeds	Pistacchi secchi
WALNUTS	2894	68.1	5.57	3.1	14.3	0.00	6.2	0	Nuts and seeds	Noci, secche
BUTTER	3117	83.4	48.78	1.1	0.8	0.02	0	7	Oils and Fats	Burro
MARGARINE	3067	82.8	42.59	0.2	0	1.70	0	680	Oils and Fats	Margarina Panetto Vegetale
MAYONNAISE	2697	70	8.82	2.2	4.2	1.13	0	450	Oils and Fats	Maionese
OLIVE OIL	3700	100	16.16	0	0	0.00	0	0	Oils and Fats	Olio d’Oliva
PALM OIL	3696	99.9	47.1	0	0	0.00	0	0	Oils and Fats	Olio di Palma
PEANUT BUTTER	2635	53.7	10.62	6.7	22.6	0.88	6.8	350	Oils and Fats	Burro di Arachidi
PEANUT OIL	3700	100	19.39	0	0	0.00	0	0	Oils and Fats	Olio di semi di Arachidi
RAPESEED OIL	3696	99.9	6.31	0	0	0.00	0	0	Oils and Fats	Olio di Colza

SOYBEAN OIL	3696	99.9	14.02	0	0	0.00	0	0	Oils and Fats	Olio di soia
SUNFLOWER OIL	3696	99.9	11.24	0	0	0.00	0	0	Oils and Fats	Olio di semi di Girasole
BACON	1142	23.6	7.97	0	15.8	3.15	0	1260	Processed Meat	Pancetta Affumicata o Bacon
PORK HAM	1183	19.7	6.49	0	26.6	6.43	0	2571	Processed Meat	Prosciutto crudo NS
PORK SAUSAGES	1259	26.7	9.44	0.6	15.4	2.75	0	1100	Processed Meat	Salsiccia di Suino, fresca
BEAN (FRESH)	526	0.6	0.12	1.3	6.4	0.01	10.6	2	Pulses	Fagioli
BEANS: GIGANTE/BUTTER	207	0.2	0.03	0.4	5.4	0.05	5.1	18	Pulses	Fave
BEANS: GREEN	101	0.1	0.02	2.4	2.1	0.01	2.9	2	Pulses	Fagiolini
BEANS: PINTO USA (DRIED)	1457	2.5	0.5	4	23.6	0.01	17	4	Pulses	Fagioli secchi
CHICK PEAS (DRIED)	1531	4.9	0.63	3.7	21.8	0.02	13.8	6	Pulses	Ceci, secchi
LENTILS (DRIED)	1491	2.5	0.34	2.4	25	0.02	13.7	8	Pulses	Lenticchie, secche
PEAS	366	0.2	0.08	4.4	7	0.00	5.2	1	Pulses	Piselli
SOY BURGER	1318	1.2	0.14	9.9	47	0.05	17.5	20	Pulses	Soia, bistecca
SOYBEAN	1765	18.1	2.54	11	36.9	0.01	11.9	4	Pulses	Soia, semi
TEMPE'	724	6.4	0.92	0.9	20.7	0.02	4.1	6	Pulses	Tempeh
TOFU	336	4.8	0.07	0.7	8.1	0.02	1.2	7	Pulses	Tofu
CHOCOLATE	2319	36	21	54	7	0.16	1	66	Sweets and Desserts	Average of Milk and Dark chocolate §
DARK CHOCOLATE	2275	34	20	57	6	0.03	1	11	Sweets and Desserts	Cioccolato fondente
HAZELNUT COVERED WITH CHOCOLATE	2387	40	20	46	9	0.27	1	109	Sweets and Desserts	Cioccolato alla nocciola
HONEY	1295	0.0	0.0	80.3	0.6	0.03	0.0	11.0	Sweets and Desserts	Miele
ICE CREAM	858	10.9	6.8	23.8	3.8	0.20	0.4	79.0	Sweets and Desserts	Average of Gelato al cioccolato, alla frutta e fior di latte †
MILK CHOCOLATE	2362	37.6	21.9	50.8	8.9	0.30	0.8	120.0	Sweets and Desserts	Cioccolato al latte
CASSAVA	1554	0.2	0.1	0.0	0.6	0.01	0.4	4.0	Tubers	Tapioca
POTATOES	340	0.1	0.2	0.4	2.1	0.02	1.6	7.0	Tubers	Patate
STARCHY ROOTS	762	0.2	0.1	2.0	1.3	0.04	1.4	17.0	Tubers	Average of Potatoes, Sweet potatoes and Cassava §
SWEET POTATO	391	0.3	0.1	5.7	1.2	0.10	2.3	40.0	Tubers	Patate, dolci
ARTICHOKE	137	0.2	0.1	1.9	2.7	0.33	5.5	133.0	Vegetables	Carciofi
ASPARAGUS	138	0.2	0.1	3.3	3.6	0.01	2.1	2.0	Vegetables	Asparagi
BEETROOT	99	0.0	0.0	3.8	1.0	0.15	2.6	58.0	Vegetables	Rape
BRASSICAS	122	0.2	0.0	2.7	2.8	0.04	2.8	14.3	Vegetables	Average of Cauliflower, Broccoli, Cabbage §
BROCCOLI	140	0.4	0.1	3.1	3.0	0.03	3.1	12.0	Vegetables	Broccoli
CABAGGE	103	0.1	0.0	2.5	2.1	0.06	2.9	23.0	Vegetables	Cavolo verde

CARROT	165	0.0	0.0	7.6	1.1	0.24	3.1	95.0	Vegetables	Carote
CAULIFLOWER	124	0.2	0.0	2.4	3.2	0.02	2.4	8.0	Vegetables	Cavolfiore
COCUMBER	64	0.5	0.2	1.8	0.7	0.03	0.6	13.0	Vegetables	Cetrioli
EGGPLANT	85	0.1	0.0	2.6	1.1	0.07	2.6	26.0	Vegetables	Melanzane
FENNEL	54	0.0	0.0	1.0	1.2	0.35	2.2	141.0	Vegetables	Finocchi
GARLIC	190	0.6	0.1	8.4	0.9	0.01	2.3	2.0	Vegetables	Aglio
GERKIN	70	0.1	0.0	2.4	0.9	1.73	1.2	690.0	Vegetables	Cetriolini sott'aceto
LETTUCE	93	0.4	0.1	2.2	1.8	0.02	1.5	9.0	Vegetables	Lattuga
MUSHROOMS	98	0.4	0.1	1.0	2.3	0.01	1.7	5.0	Vegetables	Funghi Prataioli
OLIVES	804	20.1	2.8	0.9	1.2	0.14	3.4	54.0	Vegetables	Average of Olive Nere e Verdi †
ONION	121	0.1	0.0	5.7	1.0	0.03	1.1	10.0	Vegetables	Cipolle
PUMPKIN	82	0.1	0.1	2.5	1.1	0.00	0.5	1.0	Vegetables	Zucca Gialla
RADISH	56	0.1	0.0	1.8	0.8	0.15	1.3	59.0	Vegetables	Ravanelli
ROOT VEGETABLES	195	0.1	0.1	4.2	1.2	0.11	2.0	44.8	Vegetables	Average of Potatoe, Sweet potatoe, Onion, Beetroot, Carrot, Radish §
SPINACH	147	0.7	0.1	0.4	3.4	0.25	1.9	100.0	Vegetables	Spinaci
TOMATO	88	0.2	0.0	3.5	1.0	0.02	0.9	6.0	Vegetables	Pomodori
ZUCCHINI	59	0.1	0.0	1.3	1.3	0.00	1.3	1.0	Vegetables	Zucchine

Abbreviations: SFAs= Saturated Fatty Acids. † Average values calculated from food items on the BDA-IEO database not singularly present in the study. § Average values calculated from food items on the BDA-IEO database singularly included in the final list #
