# Supplementary material for: How Much Do Front-Of-Pack Labels Correlate with Food Environmental Impacts?
Source: Nutrients. 2023 Feb 26;15(5):1176. doi: 10.3390/nu15051176 (PMC10005439; doi:10.3390/nu15051176)
Supplement: Supplementary file 1 [file nutrients-15-01176-s001.zip › Table S2.pdf]

Table S2. Correlation matrix between food scales and environmental impacts divided by food categories.

Food Scale	Food Category	GWP	Land Use	GHG	Acidification	Eutrophication	H20 withdrawal	CO2 Footprint	H2O Footprint	Sust MPI
Med Diet	Bakery products							0.886*	0.866*	0.866*
	Eggs									
	Fish and Seafood									
	Fruits									
	Grains									
	Meats	0.683	0.775	0.775	0.775	0.775	0.775	0.878	0.866	0.866*
	Milk Alternatives									
	Milk and Dairys							-0.052		-0.165
	Nuts and seeds									
	Oils and Fats									
	Processed Meat									
	Pulses	0.756*						0.247		0.437
	Sweets and Desserts									
	Tubers									
	Vegetables	-0.047	-0.258	-0.258	-0.258	-0.258	-0.775	-0.02	0.347	0.285
ITA GL	Bakery products							-0.442	-0.208	-0.061
	Eggs									
	Fish and Seafood									
	Fruits							-0.311	-0.252	-0.302
	Grains		-0.866		0.866			-0.062	0.248	-0.096
	Meats	-0.289	-0.775	-0.775	-0.775	-0.775	-0.775	-0.722	-0.735	-0.546
	Milk Alternatives									
	Milk and Dairys	-0.800						-0.607*	-0.577*	-0.576*
	Nuts and seeds							0.137	-0.577	-0.548
	Oils and Fats		0.707		0.707	0.354	0.707	0.283	0.577	0.347
	Processed Meat									
	Pulses	-0.889*						-0.642	-0.945*	-0.897*
	Sweets and Desserts									-0.845*
	Tubers									

	Vegetables							-0.117	0.310	0.344
EAT LANCET Diet	Bakery products									
	Eggs									
	Fish and Seafood									
	Fruits									
	Grains									
	Meats	-0.683	-0.775	-0.775	-0.775	-0.775	-0.775	-0.878*	-0.891*	-0.866*
	Milk Alternatives									
	Milk and Dairys	-0.872						-0.788*	-0.569	-0.713*
	Nuts and seeds									
	Oils and Fats		0.707	-0.707	0.354	0.707	0.707	-0.539	0.134	-0.134
	Processed Meat									
	Pulses	-0.791*						-0.797*	0.393	-0.454
	Sweets and Desserts									
	Tubers									
	Vegetables							0.387		0.259
Nutriscore	Bakery products							0.687	0.575	0.601
	Eggs									
	Fish and Seafood	-0.394						-0.341		-0.398*
	Fruits	-0.187						0.353	0.378*	0.387*
	Grains							0.175	-0.274	0.058
	Meats	0.356	0.632	0.105	-0.105	0.105	0.949	0.490	0.446	0.514
	Milk Alternatives							-1.000		-0.738
	Milk and Dairys	0.949						0.796*	0.644*	0.781*
	Nuts and seeds	0.204						0.249	0.411	-0.121
	Oils and Fats		-0.577	0.289	-0.866	-0.289	-0.289	-0.040	-0.481	-0.105
	Processed Meat									
	Pulses									
	Sweets and Desserts							0.828		0.828
	Tubers							0.866	0.500	0.316
	Vegetables	-0.173						-0.296		0.661

HSR	Bakery products							-0.573	-0.564	-0.539
	Eggs									
	Fish and Seafood	0.118						0.259	0.722	0.237
	Fruits	0.012						-0.135	-0.221	-0.085
	Grains	-0.894		-0.500		-0.500	-0.500	-0.556	0.222	-0.486
	Meats	0.000	-0.632	-0.105	0.105	-0.105	-0.949	-0.359	-0.297	-0.247
	Milk Alternatives									0.738
	Milk and Dairys	-0.051						-0.042	0.011	0.074
	Nuts and seeds	-0.060						-0.204	-0.090	0.154
	Oils and Fats		0.462	-0.872	0.410	0.616	0.205	-0.242	0.253	0.268
	Processed Meat							0.000	0.866	
	Pulses	0.733						-0.252	0.204	0.297
	Sweets and Desserts							-0.845*		-0.845*
	Tubers									-0.775
	Vegetables	0.219	0.632	0.632	0.632	0.632	0.316	0.132	0.148	0.038
Israeli WL	Bakery products							0.779*	0.866*	0.779*
	Eggs									
	Fish and Seafood	0.039						-0.360	-0.408	-0.193
	Fruits	-0.078						-0.060	0.351	0.132
	Grains		0.866		-0.866			0.667*	0.224	0.632
	Meats	0.139	0.632	0.316	0.316	0.316	0.949	0.558	0.438	0.540
	Milk Alternatives							-0.866		-0.894
	Milk and Dairys	0.949						0.814*	0.641*	0.808*
	Nuts and seeds	0.612						0.493	0.725*	0.412
	Oils and Fats							-0.110	-0.436	-0.275
	Processed Meat									
	Pulses	-0.247						-0.082	0.000	-0.058
	Sweets and Desserts							0.393		0.393
	Tubers									
	Vegetables	-0.173	-0.775	-0.775	-0.775	-0.775	-0.258	-0.296		0.162
	Bakery products							0.250	0.383	0.600

	Eggs									
	Fish and Seafood	-0.464						-0.415	-0.393	-0.414
NIB	Fruits	0.016						-0.089	0.335	0.036
	Grains	-0.410						-0.098	0.102	-0.178
	Meats	0.108	0.400	-0.200	-0.400	-0.200	0.800	0.631	0.522	0.240
	Milk Alternatives									
	Milk and Dairys	0.400						0.393	0.681	0.504
	Nuts and seeds	0.811						-0.017	0.072	0.277
	Oils and Fats		-0.300	0.800	-0.200	-0.500	-0.100	0.305	-0.072	-0.006
	Processed Meat									
	Pulses	0.275						0.109	-0.108	0.150
	Sweets and Desserts							0.058		-0.087
	Tubers									-0.800
	Vegetables	0.143						-0.094	0.055	0.234
	Bakery products							0.000	-0.019	0.112
	Eggs									
	Fish and Seafood	-0.325						-0.338	-0.598	-0.313
	Fruits	-0.083						-0.020	0.330	0.117
	Grains	-0.354						0.000	0.000	-0.087
	Meats	0.089	0.775	0.775	0.775	0.775	0.775	0.757	0.669	0.412
	Milk Alternatives							-0.866		-0.866
HCST	Milk and Dairys	0.354						-0.129	-0.077	-0.139
	Nuts and seeds							-0.435	0.577	0.522
	Oils and Fats									
	Processed Meat									
	Pulses	-0.247						0.509	-0.134	0.090
	Sweets and Desserts							-0.131		0.131
	Tubers							0.000	-0.866	-0.775
	Vegetables	-0.024						0.030	0.189	0.173

Abbreviations: ITA GL= Italian National Dietary Guidelines; HSR= Health Star rating; HCST= Health Canadian Surveillance Tool. *: $p < 0.05$
